# PD1 Expression in EGFRvIII-Directed CAR T Cell Infusion Product for Glioblastoma Is Associated with Clinical Response

**DOI:** 10.3389/fimmu.2022.872756

**Published:** 2022-05-06

**Authors:** Oliver Y. Tang, Lifeng Tian, Todd Yoder, Rong Xu, Irina Kulikovskaya, Minnal Gupta, Jan Joseph Melenhorst, Simon F. Lacey, Donald M. O’Rourke, Zev A. Binder

**Affiliations:** ^1^ GBM Translational Center of Excellence, Perelman School of Medicine, University of Pennsylvania, Philadelphia, PA, United States; ^2^ Center for Cellular Immunotherapies, Perelman School of Medicine, University of Pennsylvania, Philadelphia, PA, United States; ^3^ Department of Neurosurgery, Perelman School of Medicine, University of Pennsylvania, Philadelphia, PA, United States; ^4^ Warren Alpert Medical School, Brown University, Providence, RI, United States; ^5^ Department of Pathology and Laboratory Medicine, Perelman School of Medicine, University of Pennsylvania, Philadelphia, PA, United States

**Keywords:** GBM, glioblastoma, CAR T cells, immunotherapy, PD-1

## Abstract

The epidermal growth factor receptor variant III (EGFRvIII) has been investigated as a therapeutic target for chimeric antigen receptor (CAR) T cell therapy in glioblastoma. Earlier research demonstrated that phenotypic and genotypic characteristics in T cells and CAR T product predicted therapeutic success in hematologic malignancies, to date no determinants for clinical response in solid tumors have been identified. We analyzed apheresis and infusion products from the first-in-human trial of EGFRvIII-directed CAR T for recurrent glioblastoma (NCT02209376) by flow cytometry. Clinical response was quantified *via* engraftment in peripheral circulation and progression-free survival (PFS), as determined by the time from CAR T infusion to first radiographic evidence of progression. The CD4^+^CAR T cell population in patient infusion products demonstrated PD1 expression which positively correlated with AUC engraftment and PFS. On immune checkpoint inhibitor analysis, CTLA-4, TIM3, and LAG3 did not exhibit significant associations with engraftment or PFS. The frequencies of PD1^+^GZMB^+^ and PD1^+^HLA-DR^+^ CAR T cells in the CD4^+^ infusion products were directly proportional to AUC and PFS. No significant associations were observed within the apheresis products. In summary, PD1 in CAR T infusion products predicted peripheral engraftment and PFS in recurrent glioblastoma.

## Introduction

Glioblastoma (GBM) is the most common primary brain tumor in adults and a near uniformly fatal disease, with a median survival rate of under 2 years (1). The current standard-of-care for GBM involves maximal safe surgical resection, followed by chemoradiation and adjuvant temozolomide, but the malignancy is surgically incurable due to its invasive nature (2). Additional challenges for this paradigm include patients with significant residual disease following surgery, especially in the setting of multifocal disease or tumor in eloquent locations, mediating sensorimotor and language functions, or with variants like unmethylated O^6^-methylguanine methyltransferase (MGMT), which confers resistance to radiation therapy and temozolomide (3, 4). Immunotherapeutic approaches, such as chimeric antigen receptor (CAR) T cell therapy, may offer an additional avenue for treating GBM. Advantages of CAR T immunotherapy include the autologous nature of CAR T development, tumor specificity, and bypassing the requirements for antigen presentation and co-stimulatory signals necessary for an endogenous antitumor response. Early studies have assessed the efficacy of CAR T therapy in GBM patients against targets including the epidermal growth factor receptor (EGFR) variant III (EGFRvIII) (5, 6), ERBB2/HER2 (7), and IL13Rα2 (8).

EGFRvIII is a common tumor-specific splice variant of EGFR present in human tumors, found in 30% of newly diagnosed GBM cases and second in EGFR alteration frequency only to wild-type EGFR amplification (9, 10). EGFRvIII results in a constitutively active receptor that is resistant to EGFR inhibitors, and has been accordingly characterized as a negative prognostic marker (10, 11). Given the extracellular location of the alteration combined with the presence of a novel glycine residue as a result of the abnormal splicing, EGFRvIII has been an attractive antigen for immunotherapy, despite the lack of uniform expression on all glioblastoma cells. Early immunotherapeutic approaches have included monoclonal antibodies and rindopepimut, a peptide vaccine (12, 13). Beyond these approaches, our group recently concluded a first-in-human study of CAR T cells directed against EGFRvIII for recurrent GBM, which enrolled 10 patients (5). All ten patients had IDH wild-type GBM with an unmethylated MGMT promoter. This Phase I trial (NCT02209376) verified successful on-target activity and significant EGFRvIII antigen reduction in the brain following a single intravenous dose, with one patient exhibiting residual stable disease for 18 months and an overall survival of nearly 3 years (14). However, patients exhibited variable levels of CAR T trafficking to active tumor sites, changes in tumor antigen expression, and lymphocytic infiltration, suggesting significant interpatient heterogeneity in response.

Due to the autologous nature of CAR T cell development, CAR T cells are currently developed *via* T cells derived from the same patient. Accordingly, earlier work on CAR T therapy in the setting of hematologic malignancies hypothesized that differences in therapeutic success may be attributed to baseline interpatient variation in immune system deficiencies and intrinsic T cell characteristics. These variations include elevated frequencies of certain T cell populations that may be associated with higher likelihood of response (15, 16). For example, Fraietta et al. determined that remission following CD19-directed CAR T therapy for chronic lymphocytic leukemia was associated with factors such as enrichment in memory-related genes and STAT3 pathway activity at the pre-CAR T treatment baseline (15). These considerations also hold relevance in the setting of GBM, due to extensive documentation of lymphopenia even in treatment-naïve patients and GBM-induced mechanisms of T cell dysfunction, such as senescence, anergy, and exhaustion (17, 18). However, to date, no study has characterized how intrinsic characteristics and phenotypes of patient T cells and generated CAR T cells predict therapeutic efficacy for GBM or any other solid tumor. Accordingly, the aim of our study was to characterize T cell and CAR T cell characteristics in patient apheresis and transduction products predictive of peripheral engraftment and clinical response for EGFRvIII-directed CAR T therapy in recurrent GBM.

## Materials and Methods

### Phase I Trial Design

Patients with EGFRvIII-positive GBM were enrolled in a phase I open-label trial (NCT02209376), with the primary endpoints being safety and feasibility. Response rate and overall survival served as secondary endpoints. As described earlier, GBM patients were offered testing for the EGFRvIII mutation *via* a validated RNA-based next generation sequencing assay, with positive expression being defined as a minimum of 100 positive reads and patients with greater than 30% EGFRvIII expression being prioritized for enrollment (5). Across a protocol accrual period of 20 months, GBM specimens from 369 patients were tested for EGFRvIII, with 79 (21%) testing positive. Of 17 patients who consented for leukapheresis, 3 patients withdrew due to clinical decline before leukapheresis, 1 patient withdrew due to clinical decline before CAR T infusion, and 3 patients with MGMT promoter-methylated GBM did not progress to the treatment step of the protocol. 7 patients with imaging findings suggestive of GBM progression, as defined by their neuroradiology and clinical team, underwent an operation for recurrent GBM following CAR T infusion, allowing for analysis of CAR T trafficking to the brain and persistence.

### CAR T Manufacturing and Infusion

EGFRvIII-directed CAR T cells were manufactured autologously from patient apheresis products *via* the Cell and Vaccine Production Facility at the University of Pennsylvania, following validation of target specificity and efficacy against EGFRvIII-positive tumor cells *in vitro* and in xenogeneic mouse models (19). Per trial protocol, patients were required to be on 4 mg/day or less of dexamethasone for at least 5 days prior to their apheresis. Manufacturing practices followed established methods for CAR T cell stimulation, transduction, and formulation (5, 20, 21). Peripheral blood T cells from the patient apheresis product were first enriched by mononuclear cell elutriation then activated using anti-CD3/anti-CD28 monoclonal antibody-coated magnetic beads (Life Technologies) at a cell: beads ratio of 1:3. On the following day, these cells were transduced with a lentiviral vector of humanized anti-EGFRvIII single-chain variable fragment combined with the hinge and transmembrane domain of CD8 and the human 4-1BB and CD3ζ signaling domains and maintained in static culture up to day 5. On day 5, cells were transferred into perfusion bags and loaded onto the WAVE Bioreactor (GE Healthcare Life Sciences). Cells were maintained and media replenished up to day 9 when the cells were harvested. On the day of the harvest, cells were washed, debeaded, counted, and samples were removed for completing the release testing for sterility, purity, and identity. The clinical target dose of 1-5x10^8 CART cells was cryopreserved in infusible cryomedium until the patient was eligible for treatment following evidence of disease recurrence or progression, and was administered by a single intravenous infusion.

### Processing of Patient Peripheral Blood Samples

As described earlier (5), after confirmation of EGFRvIII-positive GBM and with written informed consent, leukapheresis product was obtained from the patients. CAR T manufacturing and the treatment phase of the trial began after evidence GBM recurrence or progression. Patients underwent baseline magnetic resonance imaging and subsequently received EGFRvIII-directed CAR T infusion (transduction product) within 1 week (defined as day 0). Peripheral blood samples were subsequently obtained from patients at predefined follow-up timepoints: days 1, 3, 7, 10, 14, 21, and 28. Afterwards, patients received follow-up every 4 weeks until 6 months then every 2 months until 2 years. all peripheral blood samples (apheresis, transduction, follow-up) were processed *via* the same standard operating procedures for receipt, processing, freezing, and analysis.

### Flow Cytometry

Flow cytometry analysis of patient peripheral blood samples was performed *via* the Cytek Aurora platform. Products were processed *via* a 28-marker panel including cell viability, CAR detection, T cell markers, activation markers, immune checkpoint inhibitors, and markers for other immune cell subpopulations (**Supplementary Table 1**).

Cryopreserved CAR-EGFRvIII infusion products and matched apheresis materials were thawed in complete RPMI media (RPMI 1640 supplemented with 10% FCS, 100 U/mL penicillin, 100 μg/mL streptomycin sulfate), and 0.5 U/mL benzonase (MilliporeSigma), followed by incubation at 37°C in a 5% CO2 incubator for 30 minutes. Cells were then washed with complete media without Benzonase and plated on V bottom 96-well plate. After plating, the cells were washed with PBS, stained with LIVE/DEAD Fixable Aqua (Thermo Fisher Scientific), and washed with flow buffer (PBS containing 1% BSA and 0.1% sodium azide). The cells were subsequently incubated with a surface antibodies (Abs) master mix for 20 minutes at room temperature, followed by 2 washes with flow buffer. The cells were fixed with Cytofix/Cytoperm reagents (BD Biosciences) according to the manufacturer’s instructions. Following fixation, the cells were washed twice in 1× Perm/Wash Buffer and stained with Abs against an intracellular Abs master mix for 20 minutes at room temperature. The cells were finally resuspended in PBS for acquisition on a Cytek Aurora flow cytometer. Data were analyzed with FlowJo software (Version 10).

On flow cytometric analysis, time gating was first used to gate out unstable fluidic events, and a scatter gate was applied to get rid of debris (**Supplementary Figure 1**). Next, singlet gating and live cell gating were performed. Subsequently, lymphocytes and monocytes were gated using forward scatter and side scatter plots. Following identification of lymphocytes, B cells (CD19^+^) and gamma delta T cells were next gated out. Sequentially, CAR^+^ cells and CAR^-^ cells were separated, and then each subset was divided into CD3^+^ and CD3^-^ population. After that, cells in the CD3^+^ population were further stratified as natural killer T cells (CD3^+^CD56^+^) or T cells (CD3^+^CD56^-^). Both CAR^+^ and CAR^-^ CD3^+^CD56^-^ T cells were further characterized *via* CD4^+^ and CD8^+^ staining.

CD3+ lymphocytes were classified into four subsets based on CD45RO and CCR7 positivity: effector T cells (CD45RO^-^CCR7^-^), Tem cells (CD45RO^+^CCR7^-^), naïve-like T cells (CD45RO^-^CCR7^+^), and Tcm cells (CD45RO^+^CCR7^+^).

### Statistics

Following the methods of Fraietta et al., peripheral engraftment was quantified using serial measurements of CAR T cells detected in the peripheral blood after initial infusion, *via* the area under the curve (AUC) of days following infusion plotted against log_10_ CAR copies/μg of genomic DNA at each available follow-up timepoint (15). For each patient, AUC was calculated up until most recent follow-up, even if the number of CAR T cells detected in peripheral blood transiently dropped to 0 at a single timepoint. Peripheral engraftment was calculated as overall AUC (up until the latest follow-up timepoint for the patient) and 30-day AUC (up until 30 days post-infusion). Analyzed outcomes included peripheral engraftment and PFS, as defined by the time from CAR T infusion until radiographic evidence of GBM recurrence or progression.

Before analysis, Grubbs’ test for outliers was used to assess for potential patient outliers in terms of PFS. Correlations between predictors and peripheral engraftment or PFS were analyzed *via* the Pearson correlation coefficient and, for data with a non-normal distribution, the nonparametric Spearman’s rank correlation coefficient. Statistical significance was maintained at *P*<0.05.

### Study Approval

Written informed consent was obtained prior to apheresis of enrolled patients under University of Pennsylvania Institutional Review Board protocol 820381.

## Results

### Study Subjects and Clinical Endpoints

Ten patients were enrolled in a phase 1 study (NCT02209376) for administration of EGFRvIII-directed CAR T cells to EGFRvIII-expressing recurrent GBM. One patient in the trial was excluded from our analyses due to having an outlier progression-free survival (PFS) value (PFS=615 days, p<0.001 on Grubbs’ test), leaving nine patients for analysis.

Karnofsky Performance Status (KPS) for patients preceding infusion ranged from 60–100. Seven patients underwent GBM resection following CAR infusion, allowing for analysis of changes in EGFRvIII percentage and CAR trafficking to the brain. Changes in EGFRvIII percentage post-infusion ranged from 2–53% and CAR trafficking (quantified as the ratio of concentrations in brain tumor over perihpreal blood) ranged from 0–71.177.

**Figure 1:**
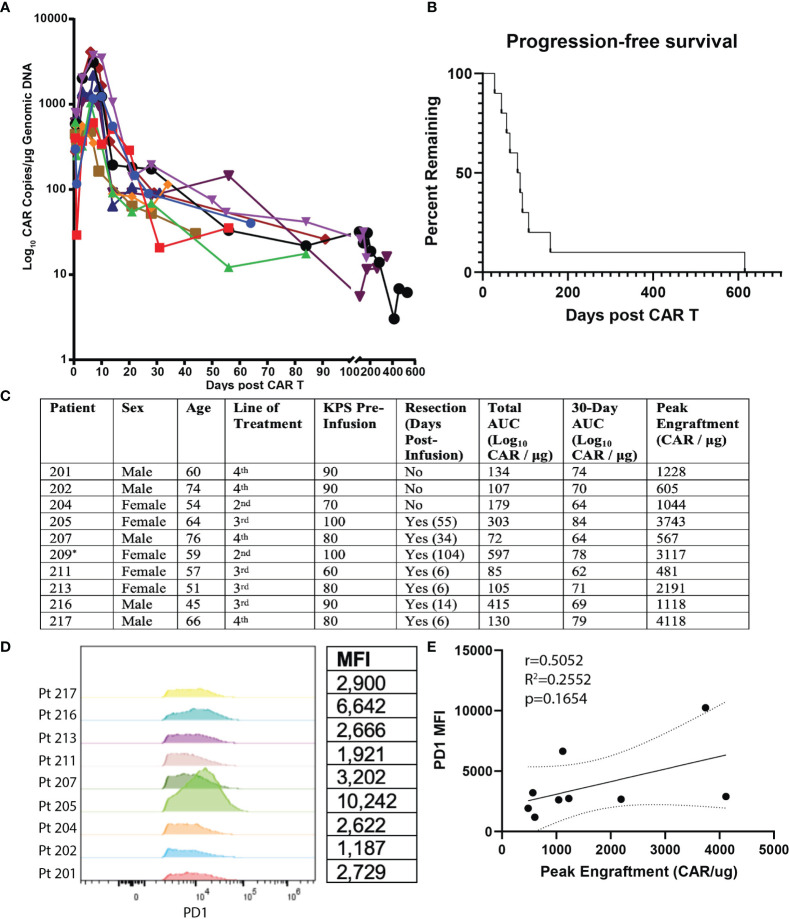
Summary of Clinical Outcomes for EGFRvIII-Directed CAR T Clinical Trial and Flow Cytometric Analysis of Patient Plasma Products. Summary of clinical outcomes for clinical trial on EGFRvIII-directed CAR T for recurrent glioblastoma (NCT02209376). Patient 209 was ultimately not included for analysis due to having an outlier PFS value. **(A)** Plot of days following CAR T infusion and engraftment in peripheral blood. Following earlier studies, peripheral engraftment was quantified as log_10_copies/μg of genomic DNA. **(B)** PFS plotted as Kaplan-Meier estimator for all subjects. **(C)** Summary values of patient characteristics, performance of resection, total AUC, 30-day AUC, and peak engraftment for all subjects. ^*^ Patient 209 was excluded due to having an outlier progression-free survival (p<0.001 on Grubbs’ test). **(D)** Comparison of PD1 expression for CD3^+^CD4^+^CAR^+^ cells for transfusion products from all nine recurrent GBM patients receiving EGFRvIII-directed CAR T. Mean fluorescence intensity (MFI) is quantified on the table to the right. **(E)** Correlation of PD1 MFI and peak CAR T engraftment levels.

The primary end points of this study were peripheral engraftment, as quantified by the AUC of log_10_ CAR copies/μg of genomic DNA measured in peripheral blood after infusion versus days in follow-up after initial infusion, and PFS (**Figures 1A–C**). Mean overall peripheral engraftment was 170 (SD=115, range=72–415) and mean 30-day peripheral engraftment was 71 (SD=7, range=64–79; **Figures 1A, C**). Mean peak peripheral engraftment was 1,677 CAR copies/μg (SD=1,379, range=481–4,118; **Figure 1C**). Median PFS was 80 days (SD=39 days) and ranged from 28-159 days (**Figure 1B**). 30-day AUC (r=0.6695, p=0.0486), total AUC (r=0.8000, p=0.0138), and peak peripheral engraftment (r=0.8167, p=0.0108) were all associated with longer PFS.

### PD1 Is Predictive of Peripheral Engraftment and PFS in Patient Transduction Products

**Figure 2:**
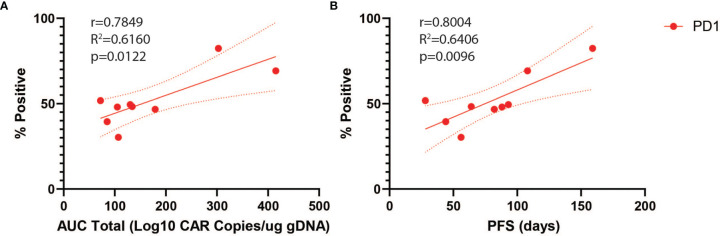
PD1 Correlations for CD4^+^CAR^+^ Cells in Patient Transduction Products. Correlation of PD1 expression with clinical outcomes for CD4^+^CAR^+^ cells in patient transduction products. **(A)** Association between PD1 expression and total AUC. **(B)** Association between PD1 expression and PFS. Error bars shown are 95% confidence intervals.

We next analyzed predictors of peripheral engraftment and PFS for CD4^+^CAR^+^ cells in patient transduction products. PD1 was the only marker in the flow cytometry panel that exhibited a significant association with peripheral engraftment and PFS in the CD4^+^CAR^+^ population and was also significantly associated with peripheral engraftment in the CD8^+^CAR^+^ population (**Supplementary Figure 2**). On flow cytometry analysis of CD3^+^CD4^+^CAR^+^ cells in patient transfusion products, mean fluorescence intensity ranged from 1,187–10,242 (**Figure 1D**). There was a positive correlation between peak engraftment and PD1 MFI (r=0.5000, p=0.1777), but this association only approached significance (**Figure 1E**). For CD4^+^CAR^+^ cells in patient transduction products, PD1 expression was positively correlated with both total AUC of peripheral engraftment (r=0.7849, p=0.0122) and PFS (r=0.8004, p=0.0096, **Figure 2**). With the addition of dexamethasone use within 5 days prior to apheresis as a confounder, the association between PD1 expression in the CD4+CAR+ population remained significantly associated with total AUC (r=0.7986, p=0.0215) and PFS (r=0.8016, p=0.0169).

Subsequently, we assessed whether these associations were also maintained for CD8^+^CAR^+^ cells in patient transduction products (**Supplementary Figure 3**). A direct correlation was also found between PD1 positivity and peripheral engraftment in the CD8^+^CAR^+^ population (r=0.6802, p=0.0438). While statistical significance was not reached when comparing PD1 expression to PFS, the trend mirrored what was seen in the CD4^+^CAR^+^ population (r=0.6363, p=0.0654).

Among seven patients undergoing reoperation after CAR T infusion, CAR T cell trafficking (ratio of CAR quantification in the brain:blood) ranged from 0-71.77 (mean=10.52, SD=26.76; **Supplementary Table 2**). However, for the infusion product, there was no significant association between PD1 expression in the CD4+CAR+, CD8+CAR+, and CD4+CAR- populations and CAR T cell trafficking to the brain (all p>0.0500). There were additionally no significant associations when this analysis was performed for an “early surgery” group of 4 patients receiving reoperation within 30 days of infusion (all p>0.0500). Notably, degree of T cell trafficking may not correlate with T cell functional activity and we are actively pursuing phenotypic characterization of clones in the infusion product.

### Association Between Immune Checkpoint Inhibitors and Clinical Response

**Figure 3:**
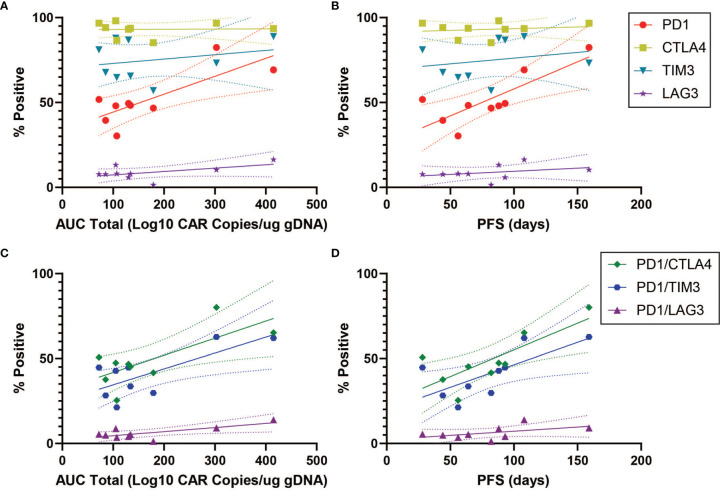
ICI Correlations for CD4^+^CAR^+^ Cells in Patient Transduction Products. Correlation of ICI expression (CTLA4, TIM3, LAG3) with clinical outcomes for CD4^+^CAR^+^ cells in patient transduction products. **(A)** Association between ICI expression and total AUC. **(B)** Association between ICI expression and PFS. **(C)** Correlation of PD1-ICI co-expression (PD1^+^CTLA4^+^, PD1^+^TIM3^+^, and PD1^+^LAG3^+^) and AUC. **(D)** Correlation of PD1-ICI co-expression and PFS. Error bars shown are 95% confidence intervals.

In order to characterize determinants of CAR T efficacy for T cells in an exhausted state, we additionally evaluated the association between immune checkpoint inhibitors (ICIs) and clinical response for CD4^+^CAR^+^ cells in transduction products. The ICIs CTLA4, TIM3, and LAG3 did not have significant associations with peripheral engraftment or PFS (**Figures 3A, B** and **Table 1**). A similar relationship was observed in the CD8^+^CAR^+^ population (data not shown). However, PD1^+^CTLA4^+^ (r=0.7338, p=0.0244), PD1^+^TIM3^+^ (r=0.7430, p=0.0218), and PD1^+^LAG3^+^ (r=0.7331, p=0.0246) co-positivity all exhibited a direct association with peripheral engraftment (**Figures 3C, D** and **Table 1**). Moreover, PD1^+^CTLA4^+^ (r=0.7739, p=0.0144) and PD1^+^TIM3^+^ (r=0.7107, p=0.0319) expression were associated with longer PFS.

**Table 1:** Summary values of CD4^+^CAR^+^ and CD4^+^CAR^-^ PD1/ICI and PD1/activation marker expression correlations in patient infusion products, associated with **Figures 2**–**5**.

		Pearson r	95% Confidence interval	R squared	p value	CAR Expression
**PD1**	**AUC Total**	0.7849	0.2522 to 0.9525	0.6160	0.0122	CAR^+^
	**PFS**	0.8004	0.2908 to 0.9562	0.6406	0.0096	CAR^+^
	**AUC Total**	0.7113	0.08950 to 0.9342	0.5060	0.0316	CAR^-^
	**PFS**	0.7260	0.1196 to 0.9379	0.5271	0.0268	CAR^-^
**CTLA4**	**AUC Total**	0.0321	-0.6458 to 0.6817	0.0010	0.9347	CAR^+^
	**PFS**	0.1763	-0.5525 to 0.7523	0.0311	0.6500	CAR^+^
	**AUC Total**	0.1418	-0.5766 to 0.7365	0.0201	0.7160	CAR^-^
	**PFS**	0.2160	-0.5232 to 0.7697	0.0467	0.5767	CAR^-^
**TIM3**	**AUC Total**	0.2502	-0.4964 to 0.7840	0.0626	0.5161	CAR^+^
	**PFS**	0.2225	-0.5182 to 0.7725	0.0495	0.5651	CAR^+^
	**AUC Total**	0.2889	-0.4643 to 0.7996	0.0835	0.4508	CAR^-^
	**PFS**	0.3091	-0.4467 to 0.8075	0.0956	0.4183	CAR^-^
**LAG3**	**AUC Total**	0.5383	-0.1959 to 0.8858	0.2897	0.1349	CAR^+^
	**PFS**	0.3366	-0.4218 to 0.8179	0.1133	0.3757	CAR^+^
	**AUC Total**	0.6018	-0.1038 to 0.9045	0.3622	0.0864	CAR^-^
	**PFS**	0.4319	-0.3256 to 0.8517	0.1866	0.2456	CAR^-^
**PD1/CTLA4**	**AUC Total**	0.7338	0.1360 to 0.9399	0.5385	0.0244	CAR^+^
	**PFS**	0.7739	0.2258 to 0.9498	0.5989	0.0144	CAR^+^
**PD1/TIM3**	**AUC Total**	0.7430	0.1556 to 0.9422	0.5520	0.0218	CAR^+^
	**PFS**	0.7107	0.08815 to 0.9340	0.5050	0.0319	CAR^+^
**PD1/LAG3**	**AUC Total**	0.7331	0.1344 to 0.9397	0.5374	0.0246	CAR^+^
	**PFS**	0.5065	-0.2375 to 0.8760	0.2566	0.1640	CAR^+^
**GRZB**	**AUC Total**	0.0400	-0.6412 to 0.6859	0.0016	0.9186	CAR^+^
	**PFS**	-0.2675	-0.7911 to 0.4823	0.0715	0.4866	CAR^+^
**HLA-DR**	**AUC Total**	-0.0293	-0.6802 to 0.6474	0.0009	0.9404	CAR^+^
	**PFS**	0.1538	-0.5684 to 0.7421	0.0237	0.6928	CAR^+^
**PD1/GRZB**	**AUC Total**	0.8217	0.3470 to 0.9613	0.6753	0.0066	CAR^+^
	**PFS**	0.7944	0.2757 to 0.9548	0.6310	0.0105	CAR^+^
**PD1/HLA-DR**	**AUC Total**	0.6237	-0.06901 to 0.9106	0.3890	0.0727	CAR^+^
	**PFS**	0.7689	0.2140 to 0.9486	0.5912	0.0155	CAR^+^

### CAR Expression Does Not Affect PD1 Correlation

**Figure 4:**
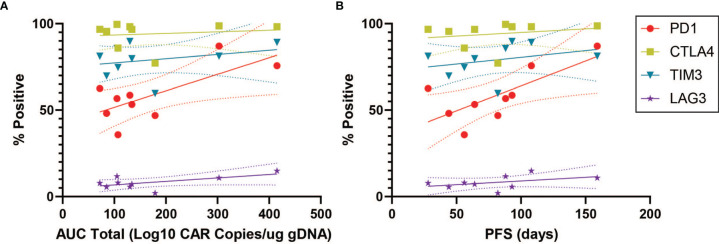
PD1 Correlations for CD4^+^CAR^-^ Cells in Patient Transduction Products. Correlation of PD1 expression with clinical outcomes for CD4^+^CAR^-^ cells in patient transduction products. **(A)** Association between PD1 expression and total AUC. **(B)** Association between PD1 expression and PFS. Error bars shown are 95% confidence intervals.

Given the association between PD1 expression in the CAR^+^ population, we subsequently analyzed this association for the CAR^-^ subpopulation. In the CD4^+^CAR^-^ population, PD1 positivity was similarly associated total AUC of peripheral engraftment (r=0.7113, p=0.0316) and PFS (r=0.7260, p=0.0268), but these associations were not observed for immune checkpoint inhibitors (**Figure 4** and **Table 1**).

### Association Between Activation Markers and Clinical Response

**Figure 5:**
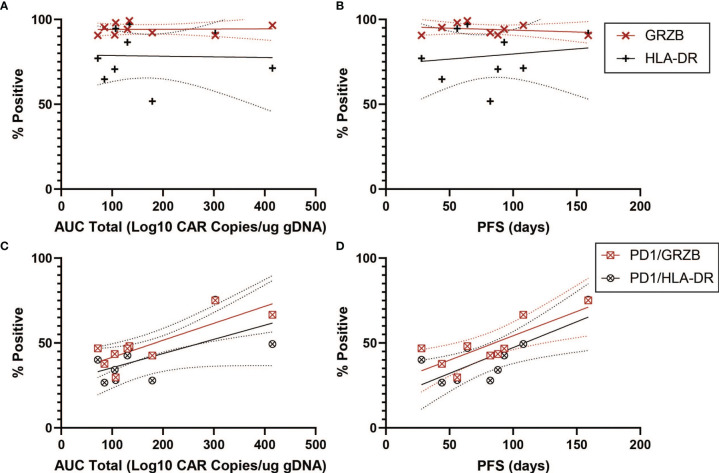
Activation Marker Correlations for CD4^+^CAR^+^ Cells in Patient Transduction Products. Correlation of activation marker expression (GRZB, HLA-DR) with clinical outcomes for CD4^+^CAR^+^ cells in patient transduction products. **(A)** Association between activation marker expression and total AUC. **(B)** Association between activation marker expression and PFS. **(C)** Correlation of PD1-activation marker co-expression (PD1^+^GRZB^+^ and PD1^+^HLA-DR^+^) and AUC. **(D)** Correlation of PD1-activation marker co-expression and PFS. Error bars shown are 95% confidence intervals.

We next turned to markers of activation in the CD4^+^CAR^+^ population in the transduction product. GRZB and HLA-DR positivity were not associated with peripheral engraftment or PFS (**Figures 5A, B** and **Table 1**). However, PD1^+^GRZB^+^ co-positivity was associated with total AUC of peripheral engraftment (r=0.8217, p=0.0066) and PFS (r=0.7944, p=0.0105; **Figures 5C, D** and **Table 1**). PD1^+^HLA-DR^+^ was also associated with PFS (r=0.7689, p=0.0155). While the association of PD1^+^HLA-DR^+^ with total AUC did not reach significance, the trend followed that of PD1^+^GRZB^+^.

### Change in PD1 Expression for Patient Apheresis Products and Association With Clinical Response

In patient apheresis products, PD1 and immune checkpoint inhibitors did not have any significant associations with peripheral engraftment or PFS (**Supplementary Figure 4** and **Supplementary Table 3A**). Mean PD1 expression for CD4^+^ cells in the apheresis product (20.6%) increased following manufacturing when compared to the CD4^+^CAR^+^ (51.7%, p=0.0002), CD4^+^CAR^-^ (58.3%, p<0.0001), and CD4^+^ (56.6%, p<0.0001) populations in the infusion product (**Supplementary Figure 5A**). However, mean PD1 expression did not change for CD8^+^ cells in the apheresis product (26.2%) in comparison to the CD8^+^CAR^+^ (13.4%, p=0.0773), CD8^+^CAR^-^ (12.8%, p=0.0656), and CD8^+^ (12.7%, p=0.0641) populations in the infusion product. There was a positive but nonsignificant correlation for initial PD1 expression in the apheresis product and post-manufacturing PD1 expression in the infusion product for CD4^+^ (r=0.4039, p=0.2810) and CD8^+^ (r=0.4680, p=0.2039) cells (**Supplementary Figure 5B**). There were no significant correlations between five manufacturing characteristics analyzed (total cell number at harvest, number population doublings, fold expansion, percent viability, and percent CAR transduction) and post-manufacturing change in PD1 expression for CD4^+^ or CD8^+^ cells (all p>0.0500).

## Discussion

Several earlier studies in the setting of hematologic malignancies have identified genotypic and phenotypic T cell characteristics predictive of CAR T clinical response, which may inform patient selection, identification of the most effective T cell populations for clinical expansion during CAR T manufacturing, and changes to transduction and activation procedures during manufacturing that may improve CAR T efficacy. However, the impact of these characteristics is less characterized for CAR T therapy for solid tumors, including GBM. In our study, we determined that PD1 expression in patient CAR transduction product was associated with peripheral engraftment and time-on-trial for EGFRvIII-directed CAR T treatment in recurrent GBM. This positive association was also observed for PD1 co-expression with ICIs (CTLA4, TIM3, LAG3) and activation markers (GRZB, HLA-DR), but was not observed for any ICI or activation marker alone, suggesting that PD1 may be the primary driver of these correlations with our surrogates of clinical response. While these correlations in the transduction product were significant across CAR^+^ and CAR^-^ subpopulations as well as CD4^+^ and CD8^+^ T cells, no significant associations were observed in the apheresis product prior to CAR T generation. The demonstration of the positive association between PD1 and our primary endpoints in both the CAR^+^ and CAR^-^ populations raises the possibility that the potential impact of PD1 on clinical response operates on the level of several cell populations in the patient infusion product as a whole, rather than just the CAR^+^ population. Moreover, the persistence of these associations in the CAR- population suggests that these findings may be partially attributable to the general manufacturing process, independent of CAR transduction. These findings collectively suggest that PD1 expression in EGFRvIII-directed CAR T transduction products may predict improved treatment response for recurrent GBM and that the differences observed were not patient-inherent but due to the infusion product preparation.

Our analysis focused on the immune checkpoint PD1, in accordance with the focus of several trials on the blockade of PD1 signaling using agents like pembrolizumab or nivolumab as an adjunct to enhance CAR T therapy (22–28), including a recently concluded trial studying EGFRvIII-directed CAR T cells in combination with pembrolizumab (NCT03726515). Our results notably contravene earlier research documenting PD1 as a marker of T cell exhaustion and demonstrating PD1 inhibition as a means of improving T cell proliferation, activation, and effector functions (24, 29–34). Consequently, several studies have investigated PD1 blockade as an adjunct to improve the efficacy of CAR T therapy against malignancies (24, 25, 32–34). Moreover, GBM has been shown to mediate PD-L1/PD1 axis signaling *via* aberrant tumor expression of PD-L1, leading to attenuation of tumor infiltrating lymphocyte response and promotion of Treg expansion (35, 36). For CAR T therapy, Finney et al. determined that PD1 levels in patient apheresis product as well as PD1 acquisition during treatment were both predictive of a worse response for anti-CD19 CAR T for acute lymphoblastic leukemia (ALL) (37). Similarly, Fraietta et al. documented poorer response for chronic lymphocytic leukemia (CLL) among anti-CD19 CAR T cells expressing PD1 (15).

Nevertheless, our present study contrarily determined that in GBM, PD1 expression was associated with an improved treatment response. In accordance with our findings, some studies have argued that PD1 is not exclusively a marker of exhaustion but may also mark other physiologic conditions such as chronic antigen stimulation or stages of T cell activation (25, 38–41). Several studies have demonstrated that PD1 is not constitutively expressed on CAR T and T cells, but is rather first induced and escalated following antigen stimulation due to its role in naïve T cell priming (28, 38, 40–42), indicating that PD1 expression may also reflect the activation status of CAR T cells, rather than exhaustion alone. It is possible that the PD1-expressing cell populations may represent an activated population of CAR T cells, rather than signifying a state of exhaustion. Moreover, Wei et al. determined that PD1 blockade actually inhibited proliferation and effector differentiation for their anti-CD19 CAR construct (41). The authors posited that PD1 may carry out several T cell regulatory functions beyond exhaustion, given the presence of two signaling motifs on the PD1 receptor. In the setting of GBM, Davidson et al. determined that PD1^+^ tumor-infiltrating lymphocytes were representative of chronically activated effector T cells with characteristics of both exhaustion and activation, and that this population had higher T cell receptor diversity and IFN-γ production than its PD1^-^ counterpart (43). In the setting of melanoma, Gros et al. additionally found that PD1 expression was a marker for clonally expanded CD8^+^ tumor-infiltrating lymphocytes (44). Finally, in a non-cancer population, Gustafson et al. demonstrated that baseline PD1 expression levels may be high even in healthy subjects and are directly correlated with the frequency of CD45RO+ memory T cells (45). These factors may explain the present study’s findings that PD1 expression was associated with improved treatment response for anti-EGFRvIII CAR T therapy. It is also important to note that these findings for our study’s anti-EGFRvIII construct may not be generalizable to other constructs. For example, earlier research has provided evidence that the addition of pembrolizumab may enhance antitumor efficacy for CAR constructs used for B-cell malignancies and malignant pleural disease (22, 25, 46, 47). However, few studies have utilized simultaneous administration of CAR T infusion and PD1 inhibition to assess the impact of PD1 modulation during the early post-infusion period, the period of peak CAR T activity in the present trial. Consequently, further functional experiments in the setting of GBM are warranted to validate these findings.

While research into determinants of peripheral engraftment and clinical response for CAR T therapy has been conducted primarily for hematologic malignancies, this study advances the understanding of these predictors in the setting of CAR T for solid tumors. Several studies have examine genotypic and phenotypic characteristics associated with the efficacy of anti-CD19 CAR T therapy for B cell malignancies, including ALL, CLL, large B cell lymphoma (LBCL), and multiple myeloma (15, 16, 37, 48, 49). Fraietta et al. and Deng et al. identified the expression of memory signatures as a positive predictor for treatment response, while markers of exhaustion signified decreased rates of treatment response (15, 48). While Fraietta et al. did not find any patient or tumor characteristics predictive of treatment response for CLL, other studies for ALL and LBCL have suggested that variables like pretreatment tumor burden are associated with clinical response (15, 37, 49). Moreover, these analyses have facilitated the identification of mechanistically relevant T cell subpopulations in apheresis or infusion products associated with treatment response (15, 16, 49). For example, enrichment of a CD27^+^CD45RO^-^CD8^+^ population in apheresis products prior to CAR T generation predicts remission for both CLL and multiple myeloma (15, 16). Nevertheless, the heterogeneity in determinants identified between studies likely emphasizes the disease- and construct-dependent nature of genotypic and phenotypic characteristics influencing CAR T therapeutic success.

The findings of the present study and the earlier body of research on predictors of treatment response raise several implications for CAR T development and utilization. Identification of T cell subpopulations associated with improved treatment response may facilitate patient selection and identification of optimal T cell populations for clinical expansion during the manufacturing process (15). These considerations are important given the immunosuppressive nature of GBM itself, with extensive documentation of T cell dysfunction in GBM patients, even in treatment-naïve status (17, 18, 36, 50). In the present study, a positive but nonsignificant correlation between baseline PD1 expression in a patient’s apheresis product and post-manufacturing PD1 expression in the infusion product was documented. However, an assessment of the association between these two variables was limited by the small sample size of the trial. Characteristics negatively influencing CAR T proliferation, engraftment, or response may also represent promising targets for modulation (15, 51). In the setting of anti-CD19 CAR T for CLL, pharmacologic inhibition of glycolysis, a pathway negatively associated with treatment response, improved CAR T proliferation and effector differentiation (15). For PD1, the phenotypic marker of focus in this study, when comparing patient apheresis and infusion products, there was a post-manufacturing increase in PD1 expression for CD4^+^ cells selectively that was not observed for the CD8^+^ population. Moreover, this change was documented across CD4^+^CAR^+^ and CD4^+^CAR^-^ cells, suggesting that the increase in PD1 expression occurred in a CAR transduction-independent manner. However, no correlations between manufacturing characteristics and change in PD1 expression with manufacturing were elucidated, which is an area warranting further study. Finally, these studies may inform clinical decisions including the optimal time during a malignancy’s course to initiate CAR T therapy. For example, Garfall et al. determined that the CD27^+^CD45RO^-^CD8^+^ T cell subpopulation predicting treatment success was significantly less enriched in relapsed cases of multiple myeloma, relative to primary cases (16). These findings hold relevance due to the present study’s focus on recurrent GBM cases, with all nine patients having received systemic therapy, including both temozolomide and radiotherapy prior to CAR T generation. Using our more recent cohort of primary GBM patients treated with EGFRvIII-directed CAR T (NCT03726515), we aim to elucidate differences in clinical response determinants between these two clinically distinct GBM subpopulations.

There are several limitations to the present study. First, this analysis is limited by the small sample size of the phase 1 study (n=9), constraining the statistical power and range of hypothesis testing that could be performed. Nevertheless, PD1 exhibited significant associations with peripheral engraftment and time-on-trial, both alone and with ICIs and activation markers, despite the small sample size of the trial. Moreover, in contrast to similar earlier analyses conducted in the setting of hematologic malignancies (15, 16, 48), there is no binary marker of clinical response for GBM that could be studied as an endpoint, leading to the use of peripheral blood engraftment and time-on-trial, as determined by clinical and radiographical findings of progression, as clinical outcomes. Nevertheless, earlier studies have determined that peripheral CAR T engraftment is a significant correlate for treatment response (49, 52), and peripheral engraftment was predictive of time until progression for our study. Additionally, this study was conducted for recurrent GBM patients, and the present findings are not generalizable to primary GBM patients, due to factors like differences in treatment history and antigen burden. Given earlier research demonstrating that systemic therapy alters patient T cell attributes (16, 53), we aim to replicate the present analyses for our follow-up trial on EGFRvIII-directed therapy for primary GBM to characterize determinants of response in this patient population. Finally, the correlations elucidated in this study alone do not ensure a mechanistic or functional link between PD1 expression and anti-EGFRvIII CAR T efficacy. Modulating PD1 activity in our CAR T construct for functional assays will be the subject of future research.

In conclusion, our study demonstrated that PD1 expression, both alone and when co-expressed with ICIs and activation markers, in patient transduction products was associated with increased peripheral engraftment and time-on-trial for recurrent GBM.

## Data Availability Statement

The raw data supporting the conclusions of this article will be made available by the authors, without undue reservation.

## Ethics Statement

The studies involving human participants were reviewed and approved by University of Pennsylvania Institutional Review Board. The patients/participants provided their written informed consent to participate in this study.

## Author Contributions

OT, LT, JM, SL, and ZB designed the research studies. OT, LT, TY, and RX conducted the experiments. OT, LT, TY, RX, and ZB analyzed data. IK and MG provided reagents. OT, LT, and ZB wrote the manuscript. All authors edited final version of the manuscript. All authors contributed to the article and approved the submitted version.

## Funding

This work was supported by funding from the Neurosurgery Research & Education Foundation (OT), the GBM Translational Center of Excellence (DO’R, ZB), The Templeton Family Initiative in Neuro-Oncology (DO’R), and The Maria and Gabriele Troiano Brain Cancer Immunotherapy Fund (DO’R).

## Conflict of Interest

DO’R receives laboratory support from Tmunity Therapeutics. DO’R and ZB are on patents relating to CAR T cell therapy for GBM.

The remaining authors declare that the research was conducted in the absence of any commercial or financial relationships that could be construed as a potential conflict of interest.

## Publisher’s Note

All claims expressed in this article are solely those of the authors and do not necessarily represent those of their affiliated organizations, or those of the publisher, the editors and the reviewers. Any product that may be evaluated in this article, or claim that may be made by its manufacturer, is not guaranteed or endorsed by the publisher.
