# Supplementary material for: PD1 Expression in EGFRvIII-Directed CAR T Cell Infusion Product for Glioblastoma Is Associated with Clinical Response
Source: Front Immunol. 2022 May 6;13:872756. doi: 10.3389/fimmu.2022.872756 (PMC9120664; doi:10.3389/fimmu.2022.872756)
Supplement: Supplementary file 1 [file DataSheet_1.pdf]

Tang et al. Supplemental Tables and Figures

Specificity	Fluorochrome	Laser/Ex Filter	Company	Cat #	Clone	Isotype	Titer (ul/100ul volume)	Notes
Viability	LIVE/DEAD™ Blue	355	Invitrogen	L34962			0.125	Stained separately
CAR EGFRvIII detection	Biotinylated EGFRvIII peptide	NA	NVS	N/A			1 uM	Stained separately
CD45RA	BUV395	355	BD Biosciences	740298	HI100	Mouse IgG2b, κ	0.1	Cell surface staining
CD16	BUV496	355	BD Biosciences	612945	3G8	Mouse BALB/c x DBA/2	0.5	Cell surface staining
CD14	BUV563	355	BD Biosciences	741360	M5E2	Mouse IgG2a, κ	2	Cell surface staining
CD28	BUV661	355	BD Biosciences	741635	CD28.2	Mouse C3H x BALB/c IgG1, κ	5	Cell surface staining
CD56	BUV737	355	BD Biosciences	741842	B159	Mouse IgG1, κ	2	Cell surface staining
CD45RO	BUV805	355	BD Biosciences	748367	UCHL1	Mouse BALB/c IgG2a, κ	0.5	Cell surface staining
PD-1	BV421	405	Biolegend	329920	EH12.2 H7	Mouse IgG1, κ	5	Cell surface staining
CD8	eFluor 450	405	Invitrogen	48-0087-42	SK1	Mouse / IgG1, kappa	2	Cell surface staining
CD19	BV480	405	BD Biosciences	746457	HIB19	Mouse IgG1, κ	0.5	Cell surface staining
CD27	BV510	405	Biolegend	302836	O323	Mouse IgG1, κ	2	Cell surface staining
CD137	BV605	405	Biolegend	309821	4B4-1	Mouse IgG1, κ	1.25	Cell surface staining
CD127	BV711	405	Biolegend	351328	A019D5	Mouse IgG1, κ	1.25	Cell surface staining
CD3	BV750	405	BD Biosciences	747058	SK7	Mouse BALB/c IgG1, κ	1	Cell surface staining
HLA-DR	BV786	405	BD Biosciences	564041	G46-6	Mouse IgG2a, κ	0.5	Cell surface staining
CD69	PerCP-Cy5.5	488	Biolegend	310925	FN50	Mouse IgG1, κ	0.5	Cell surface staining
CD95	BB700	488	BD Biosciences	566542	DX2	Mouse C3H	1	Cell surface staining
TCR gamma/delta	PerCP-eFluor 710	488	Invitrogen	46-9959-42	B1.1	Mouse / IgG1	2.5	Cell surface staining
CCR7	PE-CF 594	561	BD Biosciences	562381	150503	Mouse IgG2a	6	Cell surface staining
LAG-3	PE-Cy7	561	Invitrogen	25-2239-42	3DS223 H	Mouse / IgG1, kappa	1.25	Cell surface staining
TIM3	APC	640	Biolegend	345012	F38-2E2	Mouse IgG1, κ	2	Cell surface staining
CD4	APC-Fire810	640	Biolegend	344661	SK3	Mouse IgG1, κ	0.5	Cell surface staining
Streptavidin-PE	PE	561	BD Biosciences	554061			0.125	Cell surface staining
EOMES	FITC	488	eBioscience	11-4877-42	WD192 8	Mouse / IgG1, kappa	4	Intracellular staining
CTLA-4 (CD152)	PE-Cy5	561	BD Bioscience	555854	BNI3	Mouse BALB/c IgG2a, κ	2.5	Intracellular staining
Ki67	AF700	640	BD Bioscience	561277	B56	Mouse IgG1, κ	0.3	Intracellular staining
Granzyme B	APC-Fire 750	640	Biolegend	372210	QA16A 02	Mouse IgG1, κ	0.5	Intracellular staining

Supplemental Table 1: Flow cytometry markers

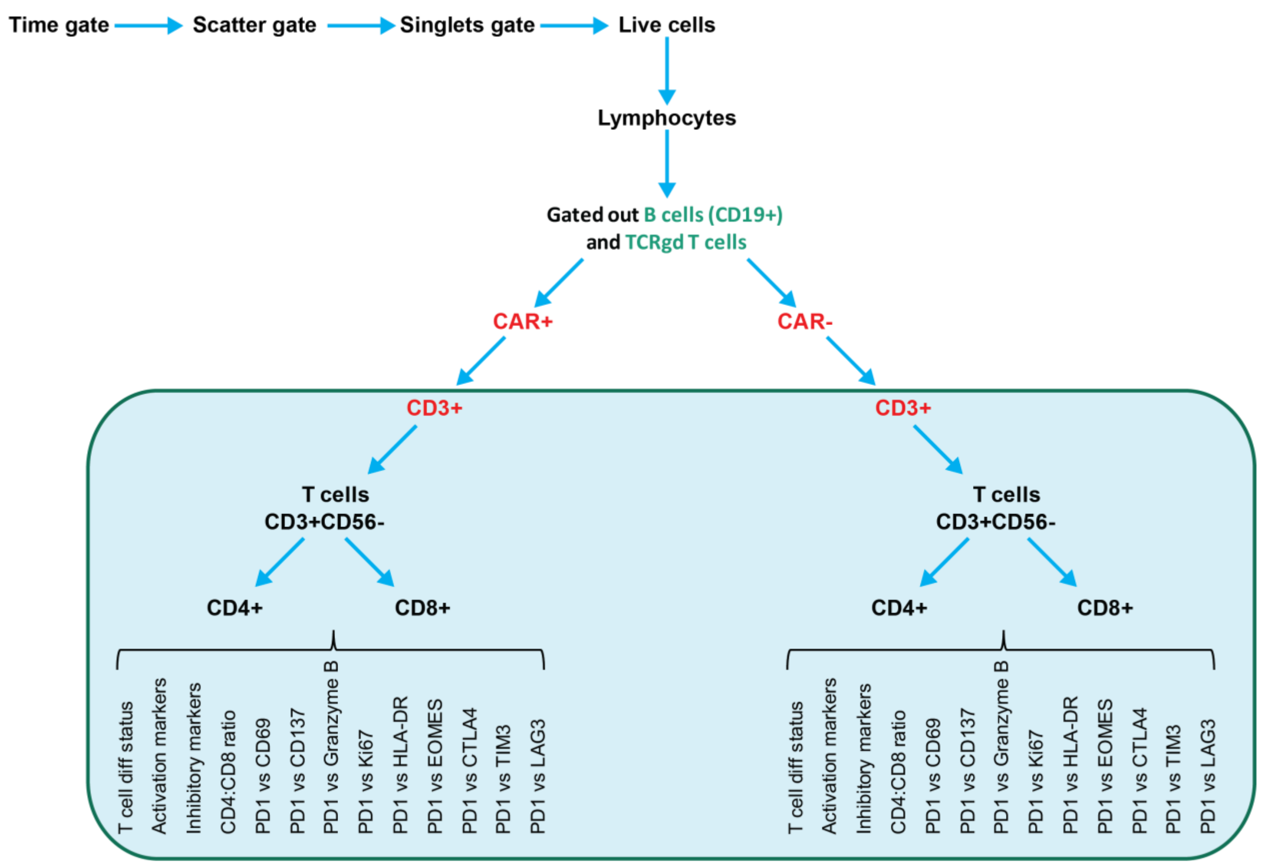

Supplemental Figure 1: Gating Strategy for Flow Cytometric Analysis. Description of methodology used on flow cytometric analysis to identify and subsequently categorize lymphocytes in patient transduction and apheresis products.

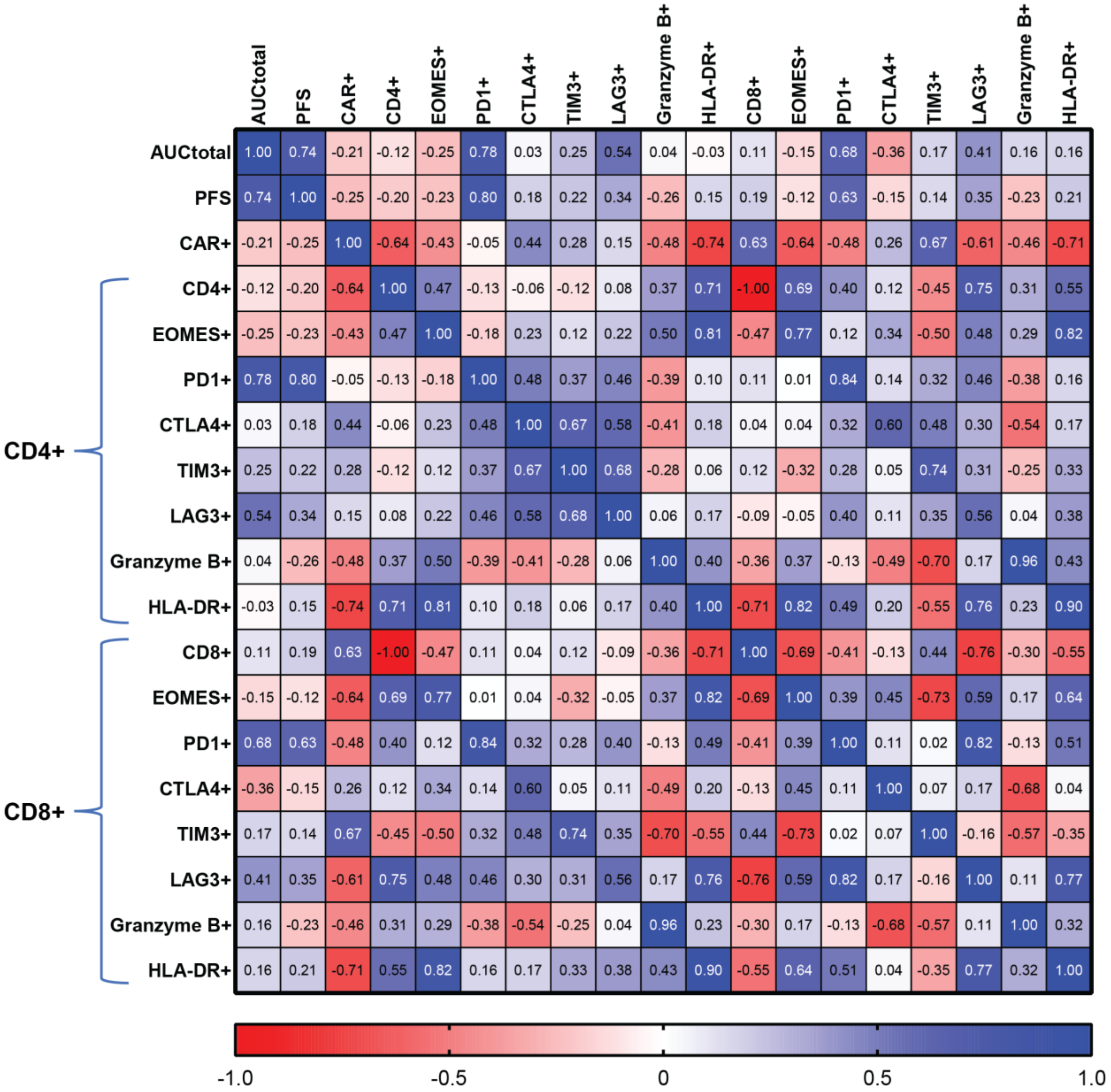

Supplemental Figure 2: Correlation Matrix for T Cell Phenotypic Markers and Clinical Outcomes. Correlation matrix for T cell phenotypic markers (CD4+, PD1, immune checkpoint inhibitors, activation markers) with peripheral engraftment and ToT. Correlations were displayed separately for $CD4^+$ and $CD8^+$ T cells.

Patient	Dexamethasone Use Before Infusion	Lymphocyte Count Before Apheresis (1,000 per mL blood)	EGFRvIII % Pre-Infusion	EGFRvIII % Post-Infusion	Ratio of CAR Quantification in Brain:Blood
201	Yes	0.5625	93%	N/A	N/A
202	No	0.5712	6%	N/A	N/A
204	No	0.7718	72%	N/A	N/A
205	No	0.7272	21%	1%	0.270
207	No	0.7620	95%	72%	0
209	No	0.7337	60%	13%	0
211	No	1.0440	42%	3%	0.079
213	Yes	0.6363	70%	40%	0.005
216	No	0.6272	96%	43%	71.177
217	No	0.3649	80%	78%	2.108

Supplemental Table 2: Patient clinical data.

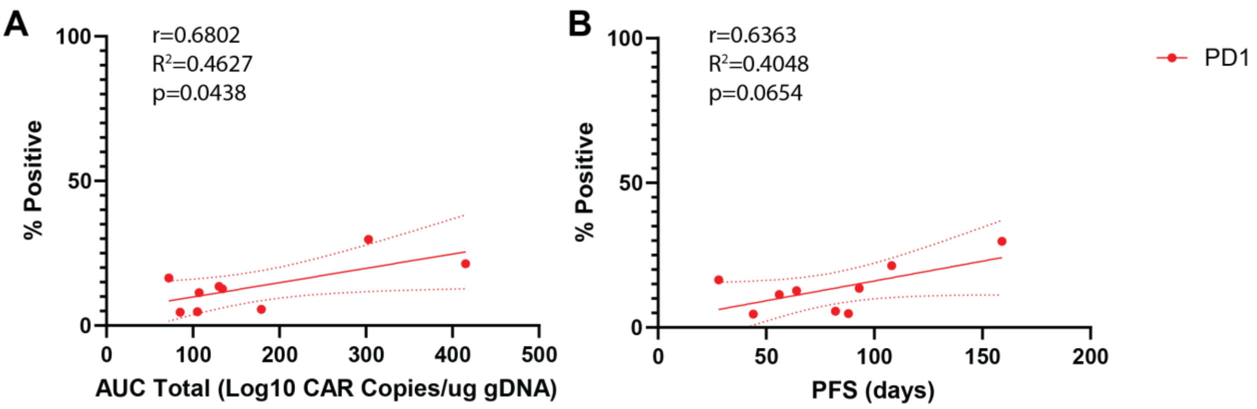

Supplemental Figure 3: PD1 Correlations for CD8$^+$CAR$^+$ Cells in Patient Transduction Products. Correlation of PD1 expression with clinical outcomes for CD8$^+$CAR$^+$ cells in patient transduction products. **A:** Association between PD1 expression and total AUC. **B:** Association between PD1 expression and ToT. Error bars shown are 95% confidence intervals.

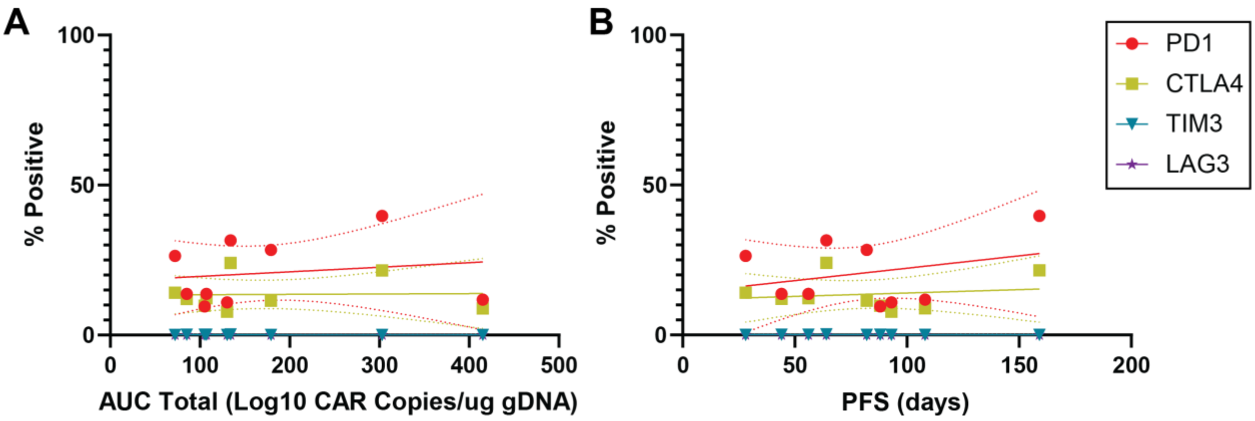

Supplementary Figure 4: PD1 and ICI Correlations for CD4$^+$CAR$^+$ Cells in Patient Apheresis Products. Correlation of PD1 or ICI expression with clinical outcomes for CD4$^+$CAR$^+$ cells in patient apheresis products. **A:** Association between PD1 or ICI expression and total AUC. **B:** Association between PD1 or ICI expression and PFS. Error bars shown are 95% confidence intervals.

		Pearson r	95% confidence interval	R squared	p value
**PD1**	**AUC Total**	0.1592	-0.5646 to 0.7446	0.0253	0.6825
	**PFS**	0.2918	-0.4618 to 0.8007	0.0852	0.4461
**CTLA4**	**AUC Total**	0.0252	-0.6498 to 0.6780	0.0006	0.9487
	**PFS**	0.1560	-0.5669 to 0.7431	0.0243	0.6886
**TIM3**	**AUC Total**	-0.3461	-0.8214 to 0.4129	0.1198	0.3615
	**PFS**	-0.1271	-0.7296 to 0.5865	0.0162	0.7446
**LAG3**	**AUC Total**	-0.2482	-0.7832 to 0.4980	0.0616	0.5196
	**PFS**	-0.1771	-0.7527 to 0.5519	0.0314	0.6485

Supplementary Table 3: Summary values of CD4$^+$CAR$^+$ PD1/ICI expression correlations in patient apheresis products, associated with Supplementary Figure 4.

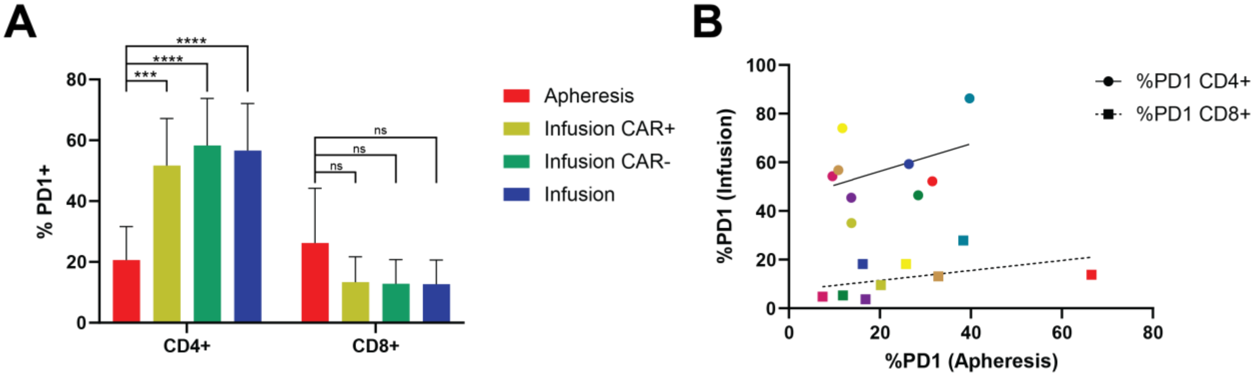

Supplementary Figure 5: Change in PD1 Expression for Patient Apheresis and Infusion Products. Change in PD1 expression for patient apheresis (pre-manufacturing) and infusion (post-manufacturing) products. **A:** Mean PD1 expression for cells in the apheresis product (red), CAR$^+$ cells in the infusion product (yellow), CAR$^-$ cells in the infusion product (green), and overall cells in the infusion product (blue). Error bars are shown for standard deviation. Expression was plotted for CD4$^+$ and CD8$^+$ cells separately. (***) denotes $p<0.0010$. (****) denotes $p<0.0001$. ns=nonsignificant. **B:** Scatter plot of PD1 expression in the apheresis product compared to expression in the infusion product for CD4$^+$ (circle) and CD8$^+$ (square) cells. Linear regression trendlines were plotted.
